# Synchronized Brain and Pulmonary Lesions: A Diagnostic Challenge

**DOI:** 10.7759/cureus.66990

**Published:** 2024-08-16

**Authors:** Nithesh Babu Ramesh, Keesari Sai Sandeep Reddy, Abinaya Srinivasa Rangan, Vimal Kumar Anandan Govindaraj, Mahendra Kumar Kalappan

**Affiliations:** 1 Internal Medicine, Saveetha Medical College and Hospital, Saveetha Institute of Medical and Technical Sciences, Saveetha University, Chennai, IND; 2 General Medicine, Saveetha Medical College and Hospital, Saveetha Institute of Medical and Technical Sciences, Saveetha University, Chennai, IND

**Keywords:** lung cancer, disseminated tuberculosis, miliary tb, daily bedside evaluation, tuberculosis, metastatic lung adenocarcinoma, diagnostic challenge, brain lesions, pulmonary lesions, synchronized lesions

## Abstract

This case report details a 50-year-old female presenting with pulmonary and neurological symptoms initially diagnosed as disseminated tuberculosis, leading to treatment with antitubercular therapy. Despite initial improvements, daily bedside evaluations revealed a new cervical lymph node, which, upon further investigation, revealed features suggestive of malignancy. Further biopsy confirmed the diagnosis of adenocarcinoma of the lung with cerebral metastases. The patient was started on palliative chemotherapy but eventually succumbed due to complications. This case underscores the diagnostic challenge of approaching synchronous cerebral and pulmonary lesions, highlighting the critical role of thorough daily evaluations in accurate diagnosis and timely intervention. While the initial diagnosis focused on tuberculosis, the discovery of the cervical lymph node was pivotal in identifying metastatic lung adenocarcinoma. This case emphasizes the importance of considering malignancy in similar clinical scenarios and using comprehensive diagnostic approaches, including advanced imaging and timely biopsies, to ensure accurate diagnosis and appropriate management.

## Introduction

In cancer diagnosis, synchronous pulmonary and brain metastases present significant diagnostic challenges [1]. These concurrent conditions can arise from various etiologies, including infectious diseases, autoimmune disorders, or cancers [2]. Understanding the complexities of these presentations is crucial for accurate diagnosis and appropriate treatment. The difficulty in distinguishing between miliary tuberculosis (TB) and metastatic lung cancer is well documented due to their overlapping clinical and radiological features. For instance, Dawlat Khan et al. reported a case where lung adenocarcinoma mimicked miliary tuberculosis, highlighting the potential for diagnostic confusion [3]. Similarly, Pitlik et al. documented cases where tuberculosis presented as malignancy, stressing the importance of including TB in differential diagnoses, especially in high-incidence regions or among high-risk populations [4]. Both conditions often present with numerous small lung nodules and can cause ring-enhancing lesions in the brain, complicating the initial diagnosis. This case report explores the diagnostic journey of a patient initially presumed to have miliary TB who was later found to have metastatic lung adenocarcinoma. It emphasizes the necessity of maintaining a broad differential diagnosis and the importance of thorough, repeated clinical evaluations. This case underscores the critical role of vigilance and comprehensive diagnostic processes in managing complex clinical scenarios.

## Case presentation

A 50-year-old female presented to our hospital with a 10-day history of low-grade intermittent fever, productive cough, involuntary movements of both upper and lower limbs, vomiting, nausea, and headache. The initial differential diagnosis included community-acquired pneumonia and acute meningoencephalitis. The patient was empirically started on intravenous antibiotics, anti-epileptic medications, and supportive care.

On examination, the patient appeared drowsy and somnolent. Her vital signs revealed a temperature of 38.6°C, a heart rate of 108 beats per minute, a blood pressure of 130/80 mmHg, a respiratory rate of 24 breaths per minute, and an oxygen saturation of 95% on room air. Respiratory system examination revealed bilateral crepitations heard on auscultation. A cardiovascular examination revealed normal heart sounds, symmetrical peripheral pulses, and no jugular venous distension or peripheral edema. Upon neurological examination, the patient had a Glasgow Coma Scale (GCS) score of 9, with equal and reactive pupils, brisk deep tendon reflexes, and bilateral extensor Babinski sign. Motor, sensory, and coordination examinations were limited due to the low GCS. The abdomen was soft, and non-tender, with no organomegaly or masses, and normal bowel sounds were heard on auscultation.

**Figure 1:**
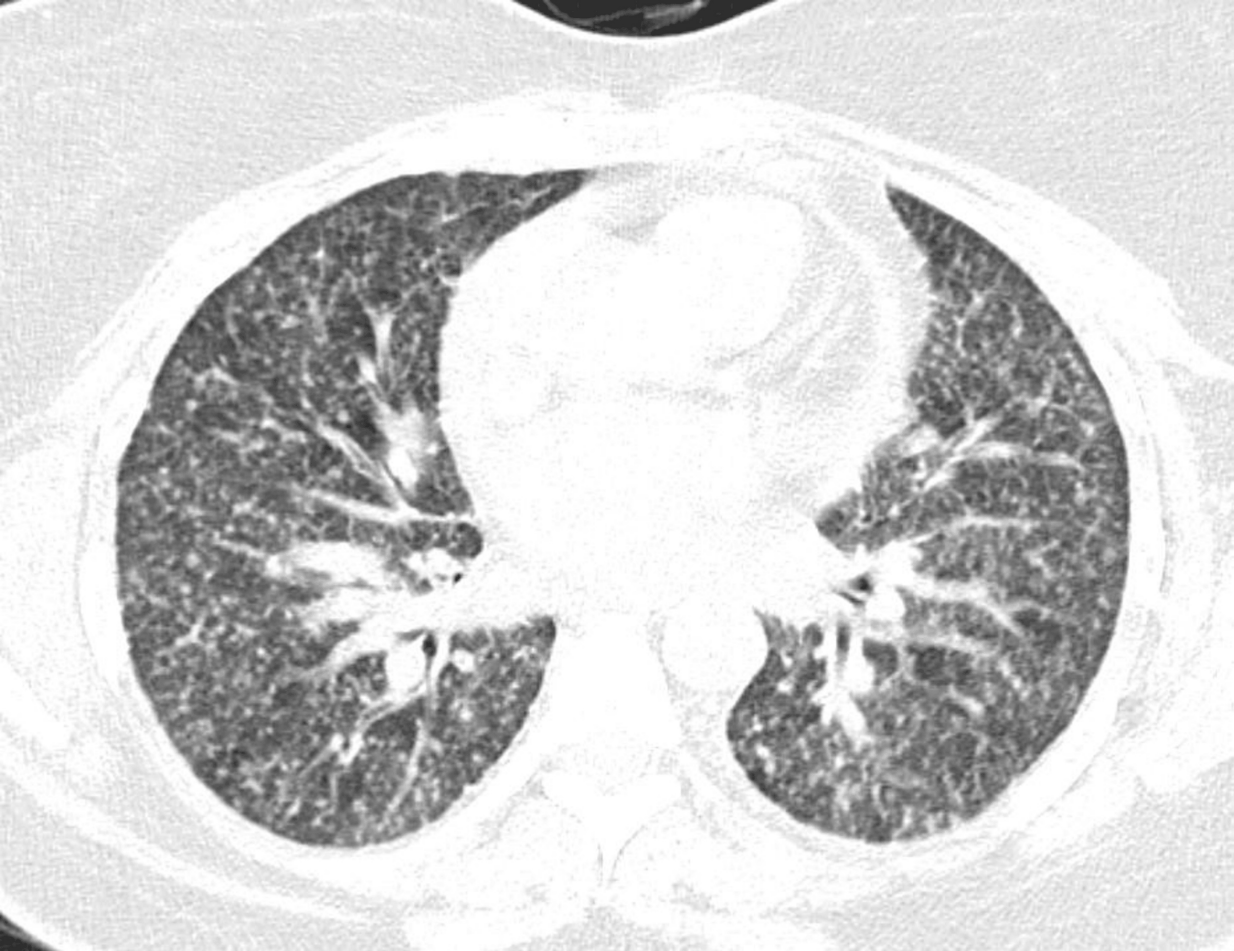
An axial CT scan of the thorax reveals multiple diffusely scattered tiny random nodules in bilateral lung fields.

**Figure 2:**
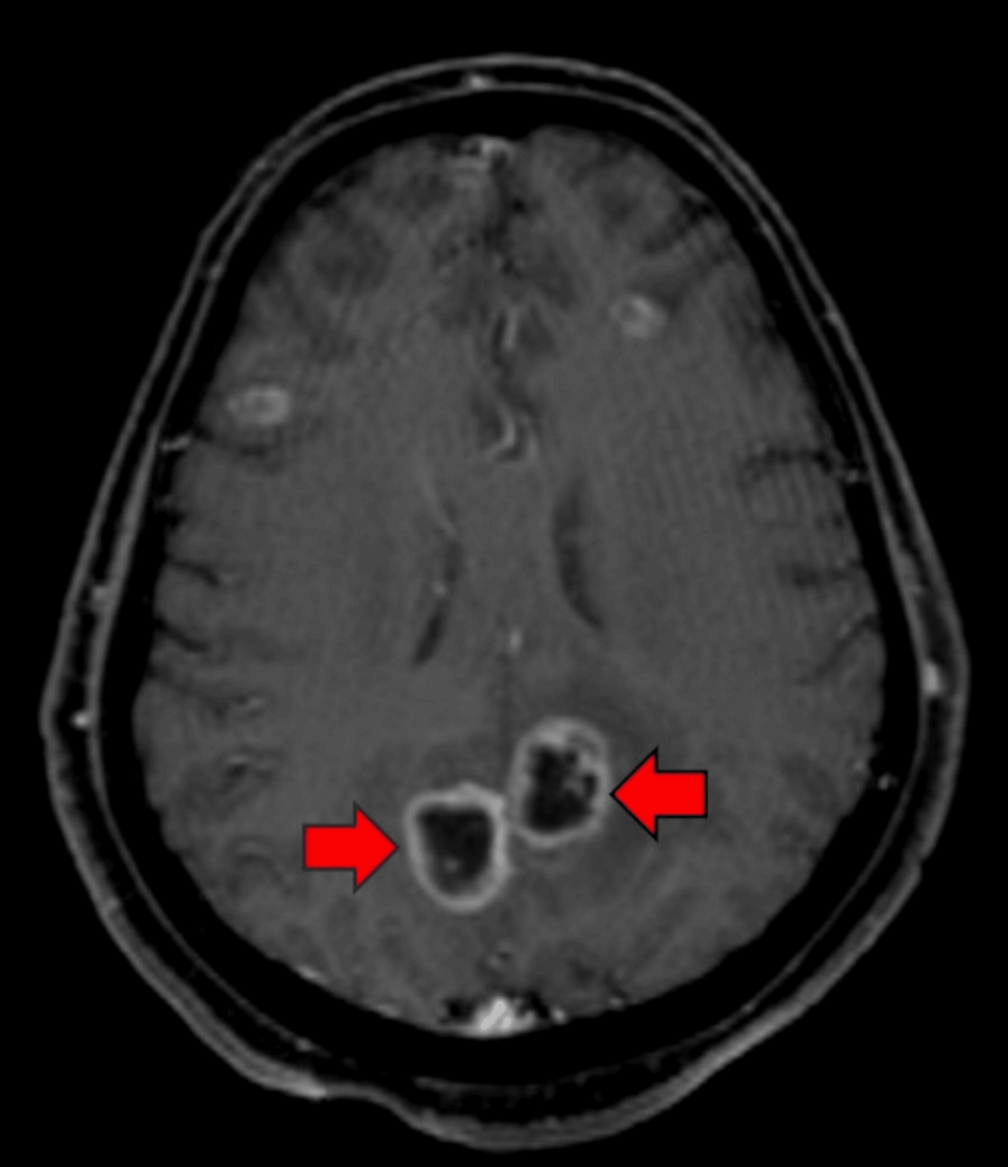
An axial MRI of the brain with contrast reveals multiple intra-axial, ring-enhancing T2 hyperintense lesions with surrounding vasogenic edema denoted by the red arrow.

Initial CT scan of the chest (Figure 1) revealed miliary pattern with features suggesting a differential of interstitial lung disease, infectious processes, or inflammatory conditions. MRI brain with contrast (Figure 2) revealed multiple ring-enhancing intracranial lesions. After analyzing the clinical and radiological picture, a preliminary diagnosis of disseminated tuberculosis was made. Sputum samples were sent for acid-fast bacilli (AFB) staining, culture, and GeneXpert Mycobacterium tuberculosis/resistance to rifampin (MTB/RIF), but the results were negative for tuberculosis. Despite negative sputum results for TB, the patient was started and continued on anti-tubercular therapy (ATT) with a regimen of isoniazid, rifampicin, pyrazinamide, and ethambutol due to the clinical response observed. The patient was planned for bronchoscopy, but the procedure was deferred on the table due to patient non-compliance.

Despite initial improvement, during daily ward rounds, we noticed a new, palpable cervical lymph node, which was further investigated. Fine needle aspiration cytology (FNAC) of the lymph node showed features suggestive of malignancy. Subsequent positron emission tomography-computed tomography (PET-CT) imaging demonstrated metabolically active lesions in the left lung, mediastinum, cervical, and supraclavicular regions, raising suspicion for a primary lung malignancy with metastases. A CT-guided biopsy of the lung lesion confirmed the diagnosis of adenocarcinoma of the lung with lymph node and neuro-parenchymal metastases. Given the advanced stage of the disease, the patient was started on palliative chemotherapy and successfully completed two cycles. However, during follow-up, the patient developed complications and unfortunately succumbed to the illness.

## Discussion

Concurrent lesions in the lungs and brain can stem from multiple etiologies, posing significant diagnostic challenges. Differentiating between infectious conditions such as tuberculosis (TB) and malignant conditions like metastatic lung cancer is critical.

Synchronous pulmonary and brain lesions can arise from various etiologies. Infectious causes include tuberculosis, which can present as miliary or diffuse lesions; mycoses such as cryptococcosis and aspergillosis; and parasitic infections like neurocysticercosis with pulmonary manifestations. Neoplastic origins often involve lung cancer metastasizing to the brain, though other cancers like breast cancer and melanoma can also metastasize to these regions. Autoimmune disorders, such as sarcoidosis and vasculitis, specifically granulomatosis with polyangiitis, can lead to such lesions. Additionally, lymphoproliferative diseases and drug-induced responses are other potential causes.

In this case, the patient's clinical history and radiological findings were suggestive of disseminated tuberculosis. The patient presented with fever, persistent cough, seizures, and other symptoms commonly observed in disseminated TB, corroborated by the radiological findings. Despite these findings, the absence of detectable TB pathogens in microbiological investigations should have prompted consideration of alternative diagnoses.

The identification of an enlarged cervical lymph node during the clinical examination was pivotal in redirecting the diagnostic approach. This underscores the importance of continually and meticulously reassessing clinical conditions in complex cases.

This case reflects the challenges described by Aung et al. [2]. Aung et al. highlight the difficulty in distinguishing between infectious and malignant etiologies in patients with synchronous lesions. They emphasize the need for a thorough, systematic diagnostic approach, including advanced imaging, histopathological evaluation, and empirical treatment trials. They stress the importance of considering a broad differential diagnosis and remaining vigilant for potential malignancies, even when infectious diseases like TB are suspected [2].

Khan et al. reported a patient initially suspected to have miliary tuberculosis based on clinical symptoms and radiological findings of diffuse nodular infiltrates in the lungs. Despite treatment for TB, the patient's condition did not improve, prompting further investigation that eventually revealed adenocarcinoma of the lung. This case, like ours, underscores the diagnostic pitfalls and the need for comprehensive tissue diagnostic workup when patients present with overlapping features of TB and malignancy [3].

Huang et al. reviewed cases of lung adenocarcinoma with synchronous brain metastases, noting the difficulties in distinguishing between infectious and malignant processes. Their findings emphasize the importance of using a combination of advanced imaging techniques and histopathological evaluation to achieve accurate diagnoses [5].

The CT findings for lung adenocarcinoma range from ground-glass nodules, part-solid nodules, and solid nodules or masses to cystic lesions, multifocal presentations, calcifications, cavitations, and appearances mimicking non-malignant processes such as infections, pneumonia, infarcts, and scar tissue [6]. This multifaceted spectrum of CT presentations complicates the diagnostic process, particularly in cases involving synchronous brain and lung lesions. The variability can obscure the diagnosis, as demonstrated in our case, where lung adenocarcinoma with brain metastases initially mimicked disseminated tuberculosis.

Jacob M et al. (2019) reported a case where epidemiological, clinical, and radiological findings initially suggested tuberculosis, but the final diagnosis was lung adenocarcinoma. Despite a high suspicion of tuberculosis, differential diagnoses for a miliary radiological pattern include metastatic disease, sarcoidosis, pneumoconiosis, and other infections. This case highlights the overlapping imaging features of tuberculosis and malignancy, with multi-organ metastases pointing towards metastatic disease. This emphasizes the need for continuous re-evaluation and consideration of other differentials [7].

The case highlights several clinical management limitations. Firstly, the delay in pursuing a tissue biopsy of the lung lesions in favor of empirical treatment may have postponed the definitive diagnosis of lung adenocarcinoma. An earlier biopsy might have identified the malignancy sooner, potentially influencing the patient's clinical course and management strategy. Secondly, the initial clinical improvement observed with anti-tubercular therapy (ATT) and steroids likely reduced the impetus for further diagnostic investigations. This over-reliance on empirical treatment, despite the lack of confirmatory evidence for tuberculosis, may have contributed to the delayed recognition of the underlying malignancy, illustrating the diagnostic challenges in regions where TB is prevalent and the differential diagnosis is broad. Lastly, the limited use of advanced imaging techniques, such as PET-CT, in the initial management approach may have delayed the identification of the malignant nature of the lesions. Early utilization of sophisticated imaging could have provided critical diagnostic insights, facilitating a more timely and accurate diagnosis.

## Conclusions

This case report highlights the diagnostic challenges in distinguishing miliary tuberculosis (TB) from metastatic lung cancer, particularly in patients presenting with pulmonary and brain lesions. The initial presentation and imaging findings of a 50-year-old female suggested miliary TB, prompting empirical anti-tubercular treatment. However, the discovery of a cervical lymph node during a routine examination proved crucial, significantly altering the diagnostic process. This case underscores the importance of considering a broad differential diagnosis, thorough and repeated clinical examinations, and the critical role of tissue diagnosis in ambiguous cases. It also highlights the utility of advanced imaging techniques, such as PET-CT, to differentiate between infectious and neoplastic changes. Emphasizing the value of a multidisciplinary approach, this case highlights the importance of ongoing critical thinking and thorough examination in complex diagnostic scenarios, advocating for the continual reassessment of initial working diagnoses due to the variable nature of diseases.
